# Arenavirus budding resulting from viral-protein-associated cell membrane curvature

**DOI:** 10.1098/rsif.2013.0403

**Published:** 2013-09-06

**Authors:** David Schley, Robert J. Whittaker, Benjamin W. Neuman

**Affiliations:** 1 The Pirbright Institute, Ash Road, Pirbright, Woking GU24 0NF, UK; 2 School of Mathematics, University of East Anglia, Norwich Research Park, Norwich NR4 7TJ, UK; 3 School of Biological Sciences, University of Reading, Reading RG6 6UB, UK

**Keywords:** virus budding, arenavirus, mathematical modelling, electromicography

## Abstract

Viral replication occurs within cells, with release (and onward infection) primarily achieved through two alternative mechanisms: lysis, in which virions emerge as the infected cell dies and bursts open; or budding, in which virions emerge gradually from a still living cell by appropriating a small part of the cell membrane. Virus budding is a poorly understood process that challenges current models of vesicle formation. Here, a plausible mechanism for arenavirus budding is presented, building on recent evidence that viral proteins embed in the inner lipid layer of the cell membrane. Experimental results confirm that viral protein is associated with increased membrane curvature, whereas a mathematical model is used to show that localized increases in curvature alone are sufficient to generate viral buds. The magnitude of the protein-induced curvature is calculated from the size of the amphipathic region hypothetically removed from the inner membrane as a result of translation, with a change in membrane stiffness estimated from observed differences in virion deformation as a result of protein depletion. Numerical results are based on experimental data and estimates for three arenaviruses, but the mechanisms described are more broadly applicable. The hypothesized mechanism is shown to be sufficient to generate spontaneous budding that matches well both qualitatively and quantitatively with experimental observations.

## 1. Introduction

Viruses are genetic parasites that epitomize the concept of the ‘selfish gene’ [1]. All viruses replicate by invading living cells, where they compete with host genes for the machinery and building blocks of life. In the process of copying itself, the virus often destroys the host cell, which can lead to disease. Viruses replicate exclusively within host cells, and onward transmission requires viral release. This is primarily achieved through two alternative mechanisms: lysis, where an infected cell dies and burst opens, so that all virions exit at once; or budding, where virions emerge gradually from a still living cell by appropriating part of the cell membrane, known as a viral envelope. Enveloped viruses cause diseases such as Ebola haemorrhagic fever, AIDS, H1N1 influenza, SARS and Lassa fever.

Recent work has even shown that hepatitis A virus, which is normally considered non-enveloped, can temporarily acquire a lipid envelope which may help the virus to spread in the presence of an immune response [2].

Most enveloped viruses share a common architecture, with at least one type of membrane-embedded, receptor-binding protein that projects out from the virion, an internal nucleic acid-binding protein that binds and protects the genome inside the particle, and a membrane-associated protein that links the internal and external virus proteins, often known as a matrix protein [3].

The development of anti-virals that interfere with the viral assembly process, known as budding, has proved challenging. In part, the difficulty in disrupting this process is due to a poor understanding of the mechanics of assembly. Determining how viral proteins force buds to form, and understanding the energies involved, are a first step in identifying potential weaknesses that could be exploited by medicines.

In eukaryotes, intracellular vesicle transport is mediated by vesicle transport proteins that are needed to move cargo between organelles and across the plasma membrane [4,5]. Four mechanisms have been proposed to explain how highly curved membranes and vesicles are formed. In the first, the membrane wraps around intrinsically curved proteins that have a high affinity for the membrane such as BAR (BIN/Amphiphysin/Rvs) domains [6] and dynamin [7]. In the second mechanism, locally high concentrations of lipid-binding protein can drive curvature by a crowding mechanism [8], although it is not clear how readily the necessary protein concentrations can be achieved in living cells. In the third mechanism, steric effects between proteins that occupy more space on one side of the membrane than the other could change the shape of the membrane [9]. In the fourth mechanism, bending is triggered by a conformational change, causing part of a protein to be inserted like a wedge in the membrane, stretching one side of the membrane more than the other and causing the membrane to curve in response. Examples of proteins that are believed to work in this manner include Sar1p [10], Epsins [11], ADP-ribosylation factors [11], which drive vesicle budding towards the cytoplasm, and the influenza virus M2 protein [12], which helps to cut new virus particles free of the cell.

Most enveloped viruses exit the cell in three steps: first, virus proteins accumulate as a raft on the membrane; second, the proteins form an outward-facing membrane bulge called a bud; and, third, the bud is snipped free from the rest of the cell membrane in a process called abscission [13]. Table 1 summarizes what is currently known about the minimal requirements for formation of virus-like particles (VLPs) of enveloped viruses. VLP formation requires both budding and scission. In most enveloped viruses, the accumulation and budding steps are driven by matrix proteins and surface glycoproteins, whereas abscission is carried out by host ESCRT proteins or virus-encoded release proteins.

**Table 1.** The role of proteins in release of enveloped viruses that infect vertebrates.

virus family	matrix	VLP formation	scission
Arenaviridae	Z	Z [14–16]	L-domain [14,16]
Bunyaviridae	^a^	GN and GC [17]	
Orthomyxoviridae	M1	M1 [18,19]	M2 [12]
		M1 NA and HA [20]	
		NA and HA [21]	
Filoviridae	VP40	VP40 [22,23]	L-domain [22]
Rhabdoviridae	M	M [24,25]	L-domain [26,27]
Paramyxoviridae	M	M [28,29]	L-domain [28]
Bornaviridae	M	M and G [30]	L-domain^b^
Coronaviridae	M	M [31], M and E [32]	
Arteriviridae	M, GP5	M GP5 and N [33]	
Flaviviridae	^a^	prM/M and E [34,35]	
Togaviridae	^a^	E2 and C [36]	
Retroviridae	Gag	Gag [37,38]	L-domain [39,40]

^a^These viruses appear to lack a discrete matrix protein, but the matrix function may be carried out by glycoprotein transmembrane and cytoplasmic tail regions.

^b^Borna disease virus M protein contains a YXXL motif that has not yet been demonstrated to function as an L-domain.

We have chosen arenaviruses as our exemplar (see the electronic supplementary material, figure S1). Arenavirus virions assemble as a flat raft inside the plasma membrane, which then forms a bud by a poorly understood mechanism that involves Z [41]. Newly formed arenavirus buds are finally cleaved free of the cell with [14,16,42] or without assistance from ESCRT proteins [15,43]. The viral nucleoprotein NP may facilitate this process in some viruses [43,44]. Z is both necessary and sufficient for the release of arenavirus-like particles [15,16,42]. Z is heavily embedded in the inner face of the virus membrane [45], in a myristoylation-dependent manner [46]. Membrane-bound Z is also required to maintain a spherical virion shape [45].

**Figure 1.**
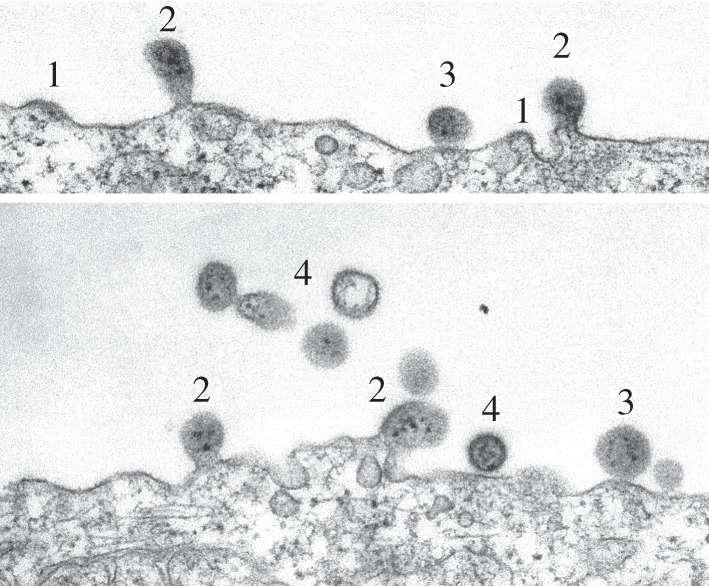
Electron micrographs of lymphocytic choriomeningitis virus emerging from an infected cell. The early budding stage is characterized by thickened membranes (1), which then bulge outwards (2), becoming spherical projections tethered to the membrane (3) and, finally, mature virions (4).

Arenavirus proteins accumulate at flat membranes before bud formation [47–50], as shown in figure 1. The accumulated flat mats of viral protein can extend over a much larger area than is required to form a virion [50]. This suggests that bud formation is unlikely to be driven directly by protein–protein crowding, asymmetric protein distribution or intrinsic curvature of the virus proteins. Although structurally distinct from other virus matrix proteins [51–53], arenavirus Z and other matrix proteins have been reported to bind the membrane deeply enough to displace an estimated 5–10% of lipid molecules from the inner membrane face of fully assembled virus particles [45]. The immersion of Z in the inner side of the virus membrane suggests that arenaviruses may bud by deforming the membrane with wedge-like amphipathic protein domains. However, Z insertion into the cytoplasmic side of the membrane would be expected to produce an inward membrane curvature, which does not occur. Because none of the four proposed mechanisms is both consistent with structural data and expected to produce an outward bud, we favour a fifth mechanism.

**Figure 2.**
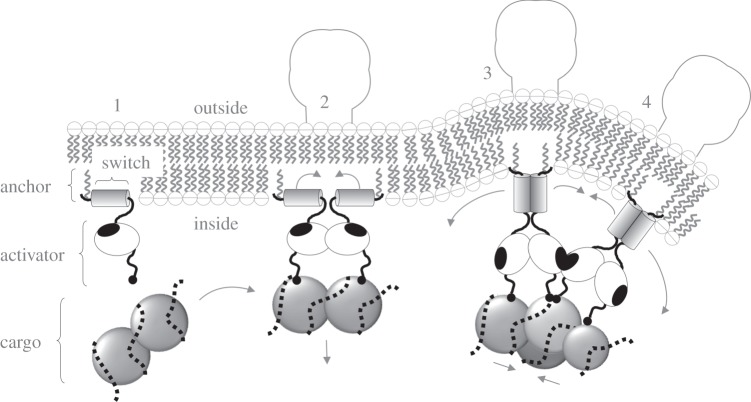
Hypothetical anchor, switch and activator model of virus budding explored in this study. Arenavirus Z is shown embedded in a lipid bilayer by means of a covalently attached myristate anchor (wavy line) at the N terminus, followed by an amphipathic switch (shaded cylinder) and a C-terminal activator (white oval with tail) that has a potential activator–activator interaction site (black oval). Immediately after translation (1), the hydrophobic side of the switch is inserted in the membrane awaiting the arrival of the virus cargo. In the context of a viral protein assembly (2)–(4), a simultaneous force applied to all the activators in the assembly exposes multiple switches, allowing the hydrophobic faces of the switches to come together in the cytosol. This reduces the available inner leaflet area leading to a bulge (3) that can be stabilized (4) by interactions between groups of proteins.

The new proposed mechanism of arenavirus budding is shown in schematic form in figure 2. The proposed mechanism involves coordinated removal of amphipathic wedges from the cytoplasmic face of the membrane. This would be energetically equivalent to a mechanism of curvature driven by amphipathic wedge insertion. While further structural characterization of pre-budding Z would be needed in order to test the validity of this mechanism, the purpose of this study is to examine the biophysical feasibility of amphipathic wedge removal as a budding mechanism for arenaviruses.

To quantify the potential change in curvature that could be induced by viral proteins, we consider a hypothesized activator model for arenavirus, as described in figure 2. It has previously been shown for a wide range of viruses that membrane lipid is displaced by virus matrix proteins [45], with significant changes in the inner leaflet but not the outer leaflet. Calculations here are therefore based on the assumption that membrane curvature is induced by an asymmetric change in the amount of space GP and Z occupy in the two membrane leaflets. Several cellular [5,10,11] and viral [12] proteins have been proposed to induce membrane curvature in a similar way by inserting amphipathic protein domains into one face of the membrane.

To show the capacity for induced curvature alone to generate recognizable buds, we model the cell membrane as a shell whose innate mean curvature 1/*r*_c_ (where *r*_c_ is the idealized cell radius) is modified in the presence of viral proteins to 1/*ρ*. For the sake of simplicity, proteins are assumed to cover an axisymmetric region on a spherical cell. This assumption is supported by electron micrographs that show that Z forms a layer along the underside of the viral membrane in round virions (see the electronic supplementary material, figure S2). While Z can be difficult to see on individual images (electronic supplementary material, figure S2 upper panels), it becomes clearly visible when hundreds of virion images of a similar size are averaged (see the electronic supplementary material, figure S2 lower panels). The use of a mathematical model allows us to also investigate the potential for interactions between the proteins to stabilize the growing bulge by making the membrane rigid, effectively locking the curvature in place. Although rigidity has not been specifically demonstrated for arenaviruses, it is known in icosahedrally ordered enveloped viruses such as alphaviruses and flaviviruses [54–56], and can be inferred for some non-icosahedral viruses from the architecture of empty filamentous influenza virus capsids and immature coronaviruses [57]. Previous observations of enveloped viruses [12] show that virions form from growing bulges that eventually form spheres attached to the membrane by a narrow tube. Cellular endosomal sorting proteins have been implicated in the abscission stage of arenavirus budding [41], and are assumed here to finish the budding process by severing the connection between the virion and the cell.

In this paper, a mechanism of viral-protein-induced budding is proposed. Experimental work indicates that membrane protein is strongly associated with membrane curvature, whereas a mathematical model shows that membrane curvature is sufficient to produce fully formed buds. Furthermore, data for arenavirus demonstrate that the proposed mechanism produces budding vesicles that are qualitatively and quantitatively consistent with observed virions.

## 2. Results

### 2.1. Viral proteins are associated with membrane curvature

**Figure 3.**
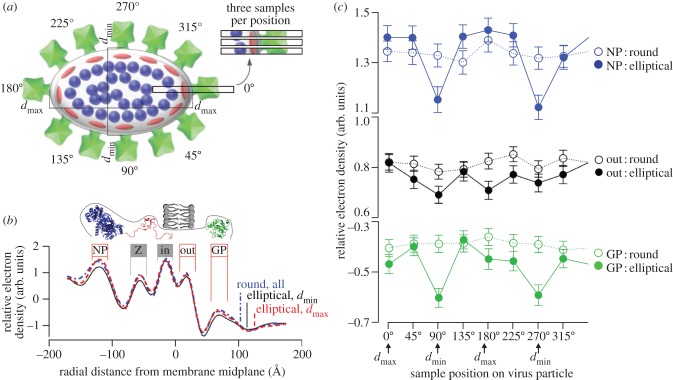
Relationship of virus proteins to local membrane curvature. (*a*) Three transects were recorded at eight positions around each virion, relative to the longest (*d*_max_) and shortest (*d*_min_) visible diameter. (*b*) Expected positions of the external viral glycoprotein (GP; square), matrix protein (Z; oval) and nucleoprotein (NP; circle), as well as the inner (in) and outer (out) phosphate groups of the Tacaribe virus membrane are indicated. Averaged electron density transects from the edge of size- and micrograph-matched round virus, the curved tips of elliptical virus and the less curved sides of elliptical virus are shown. (*c*) The average density in the GP, outer membrane and NP regions indicated in (*a*) are shown at the eight sampled positions around each virion. (Online version in colour)

Experimental measurements confirm that the presence of glycoprotein, nucleoprotein and Z are all strongly related to the curvature of the membrane (see the electronic supplementary material, figure S3). Figure 3*a* shows how density data were sampled; figure 3*b* shows average density data at eight cardinal points around each virion; figure 3*c* shows how electron density changes along the virion edge.

Results summarized in table 2 show that there is significantly less of each protein at the flatter ‘sides’ of ellipsoidal virus particles than at the curved ‘tips’ of ellipsoidal particles (GP, *p* < 10^−5^; Z, *p* < 10^−3^; NP, *p* < 10^−3^) or ‘edges’ of spherical particles (GP, *p* < 10^−5^; Z, *p* < 10^−3^; NP, *p* < 10^−3^), but no difference between ‘tips’ and ‘edges’ (GP, *p* > 0.95; Z, *p* > 0.97; NP, *p* > 0.60). Proteins appear covariant with respect to the three positions—see table 2 for explicit values—despite being poorly correlated with each other (Z–GP, *r* = 0.25; GP–NP, *r* = 0.14; NP–Z, *r* = 0.13). There was no evidence for other membrane changes between the ellipsoid tips and other positions to explain the curvature, with no significant differences found between inner face (*p* > 0.44, 0.52) and outer face (*p* > 0.13, 0.32) signal strengths.

**Table 2.** Electromicrography signal. Mean signal intensity at each position, as estimated by a mixed effects linear model, with little difference between edge and tip (−0.01 to 0.04) but a consistent drop between edge and side (−0.20 to −0.15).

position	signal intensity		
	GP	Z	NP
spherical edge	−0.41	0.77	1.30
ellipsoidal tip	−0.43	0.76	1.34
ellipsoidal side	−0.56	0.57	1.09

### 2.2. Estimated protein-induced changes in the membrane curvature and stiffness

Based on the hypothesized mechanism described in figure 2, we evaluate (4.6) using parameter values given in table 3 to obtain a quantitative estimate of the innate mean curvature 1/*ρ* in the budding region for arenavirus. We find *ρ* ≲ 5.95–21.9) × 10^−8^ m. By examining the shape of similar-sized vesicles and virions, we are able to estimate the effect of viral proteins on the stiffness of the membrane. We estimate the relative change *β* in the membrane bending stiffness in the presence of viral proteins using equation (4.8). *B*_0_ is defined as the innate bending stiffness of the (virus protein free) cell membrane with *β* given by the ratio of observed deformations between protein-free vesicles and arenavirus virions (shown in the electronic supplementary material, figure S1).

**Table 3.** Parameters. Biological parameters relevant to mechanistic model: see §§4.6 and 4.7 for details and sources.

parameter	description	value
*r*_c_	cell radius	(7.5–10)×10^−6^ m
*r_v_*	virion radius	(1.7–13.1)×10^−8^ m
*δ*	cell membrane thickness	(3.4–5.0)×10^−9^ m
*|p|*	cell pressure differential	≤O(1) N m^−2^
*B*_0_	membrane bending stiffness	(0.11–2.3)×10^−19^ N m
*H*	membrane shear modulus	(2–6)×10^−6^ N m^−1^
*D*_vesicle_	vesicle relative deformation	0.07
*D*_virion_	virion relative deformation	0.029–0.041
*a*	area removed by one Z protein	(1.44–1.80)×10^−18^ m^2^
*n*	number of proteins in group	two or four
*A*_g_	surface area per group	6.3×10^−17^ m^2^

Size was not found to be strongly correlated with shape for any of the three arenaviruses considered: Pichinde (PICV) (*|r|* < 0.01); Tacaribe (TCRV) (*|r|* < 0.01); or lymphocytic choriomeningitis (LCMV) (*|r|* < 0.1). For vesicles without viral protein, the mean ratio of each vesicle's maximum diameter to its minimum diameter was 1.070 (based on a total of *n* = 195 vesicles found in the virus preparations). For PICV, TCRV and LCMV, the mean ratio of each virion's maximum diameter to its minimum diameter was 1.029, 1.039 and 1.041, respectively (based on *n* = 2810, *n* = 1672 and *n* = 2242 virions). It follows from equations (4.7) and (4.8) that *β* ≲ 2.5.

### 2.3. Bud formation can be achieved by changes in the membrane curvature alone

Our mechanical model of the cell membrane shows that changes in the local innate curvature of the membrane are alone sufficient to drive bud formation. Our numerical solutions reveal the quasi-steady evolution of the membrane shape as the budding area *A*_p_ (that part of the membrane to which viral protein is attached) grows with time. For small values of *A*_p_, a mound forms on the cell surface. This becomes a distinct bud shape as this region increases, and eventually forms a spherical virion attached to the cell by a thin ‘neck’. For dynamic evolution, see the electronic supplementary material, video S4. These forms are comparable with real-life observations (figure 1), although the continuum model does not extend to the limit where the bud pinches off (see §4 for details).

**Figure 4.**
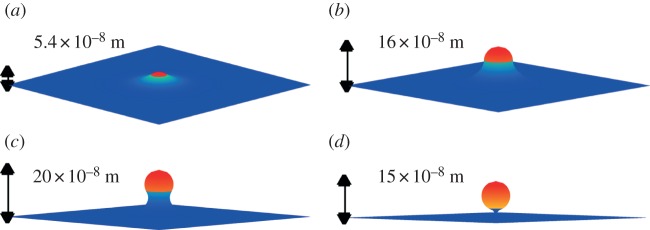
As the area of protein-bound membrane increases, the quasi-steady solutions reveal a growing bud. Here (*a*) *α* = 0.1; (*b*) *α* = 0.5; (c) *α* = 0.8; (*d*) *α* = 1.0, where *α* is the area of budding region as a fraction of the total surface area of a spherical vesicle with radius *ρ*. Other parameters as in table 3 (lower bounds, including *T*_0_ = 0) with *β* = 1 (no additional-induced stiffness). The budding region is shaded red, with the protein-free cell membrane shaded blue. For full animation, see the electronic supplementary material, video S4.

We further confirm that such behaviour is possible within realistic biological parameter ranges. As well as the change in curvature in the budding region, our model has three key parameters: the non-dimensional size *α* of the budding area; the relative change in stiffness *β* in the budding area; and the far-field tension *T*_0_ in the cell membrane (which is related to the transmembrane pressure difference *p*). Solving the model across a range of parameter values, including those presented in table 3, indicates that bud development occurs in the biologically relevant regime: figure 4 shows the numerical solution of the system as the area of viral-protein-attached membrane (the budding region) increases. With protein covering a region commensurate with the surface area of a vesicle with radius *ρ*, the steady state of the system is a distinct bud.

### 2.4. Effect of membrane stiffness on bud profile

Our numerical simulations show that variations in the membrane stiffness become more relevant to the system as the far-field tension *T*_0_ increases. It follows from equations (4.45) and (4.46) that, if *T*_0_ is negligibly small, *β* needs to be unjustifiable large to have a noticeable impact on the system. Increased stiffness of the budding region (*β* > 1) can produce a tighter bud with a smaller radius, thus increasing the potential to pinch off for smaller budding regions. Electronic supplementary material, figure S5 shows numerical solutions for the case where the transmembrane pressure *p* is of the order of 1 N m^−2^, and the area of the budding region is 4*πρ*^2^ (the surface area of a sphere of radius *ρ*), as the bending stiffness of the membrane in the budding region increases. When sufficient protein is present, a bud can be closed under any far-field tension *T*_0_, provided *β* is sufficiently large (as *β* → *∞* the budding region membrane is forced into a sphere with radius *ρ*), although this is unlikely to be biologically realistic.

### 2.5. Predicted buds match observed virions

With biologically relevant parameters for arenavirus, the numerical model predicts that the proposed protein-induced curvature mechanism will yield buds of a similar size to those observed experimentally. Using the values in table 3, the model predicts that the buds generated will be approximately spherical with radii in the range 5.95 × 10^−8^ to 21.9 × 10^−8^ m, whereas properly formed arenavirus virions are usually observed to have a radius in the range 1.7 × 10^−8^ to 13.1 × 10^−8^ m (table 3).

For the model presented, the total energy in a spherical bud with radius *ρ*, as given by equation (4.53) using values from table 3, is of the order 10^−19^ J. In the absence of all other interactions, this would be the predicted energy required to remove all the switches within the budding region: this is equivalent to the order of 10^−2^ k cal mol^−1^ (10^−22^ J) for each embedded protein group, which is well within the free energy of partitioning levels observed experimentally [58].

## 3. Discussion

The production of new virions is a critical stage in viral infection, so a better understanding of how vesicle budding occurs could help in developing treatments to potentially interrupt this process. In this paper, a viable mechanism for viral-protein-driven budding has been identified.

Viral proteins do not just lie under the membrane, but embed in it [45]. Here, it is shown that such viral proteins are significantly associated with virion membrane curvature. Interactions between the arenavirus glycoprotein, matrix and nucleoprotein have been demonstrated by cross-linking [59,60] and co-immunoprecipitation assays, but a direct relationship between protein interactions and curvature has not been demonstrated previously.

A plausible explanation of viral-protein-induced curvature is presented for the example of arenavirus. The mechanism takes account of the fact that proteins embed in the inner but not outer leaflet of the membrane [45], although the binding of genome into the bud—thereby forming a function virion—is integral to the mechanism proposed. We note, however, that this explanation is not essential for acceptance of the overall budding model, which is based on proven curvature associations, but that it helps validate the model by providing a quantitative test of predictions. Conversely, the quantitative agreement between predicted and observed results supports the hypothesis presented in figure 2.

A mechanical membrane model shows that additional-induced curvature of the cell membrane, of a magnitude consistent with known protein and cell properties, is sufficient alone to result in realistic sized buds emerging from a flat cell surface. The addition of a viral-protein-induced increase in the membrane stiffness means buds can form more quickly in the presence of significant transmembrane pressure, in the sense that fully formed buds are produced from smaller areas of viral-protein-bound membrane. Although simulations here focus on spherical buds, the membrane model is compatible with the emergence of other (e.g. filamental) shaped virus particles budding from the membrane. Indeed, we would expect this to be the case in practice, when the distribution of curvature-inducing viral protein is not expected to be uniform or restricted to a simple circular patch. The membrane model is valid only to the point of pinch off, but the mechanism whereby the connection between the virion and cell are severed (believed to be executed by cellular endosomal sorting proteins) has already been established [41]. Calculations here are based on the explicit properties and measurements of arenavirus, but the models could potentially be applied to the budding of other pleomorphic viruses.

## 4. Methods and models

### 4.1. Virus growth and preparation

Pichinde virus-AN3739 (PICV), Tacaribe virus-TRVL 11573 (TCRV) and Lymphocytic choriomeningitis virus-Arm53b (LCMV) strains were grown in baby hamster kidney cells. Virions were purified from cell culture medium by polyethylene glycol precipitation and Renografin density gradient centrifugation [59].

### 4.2. Electron microscopy

Low-dose cryoelectron microscopy of purified arenaviruses was performed at 100 kV, and images were recorded on film. Micrographs were digitized by using a Zeiss SCAI scanner with Phodis software. Images were scanned at a resolution of 4 × 10^−10^ m per pixel at the level of the specimen. The histogram for a representative portion of the image containing vitreous ice and protein was normalized by adjustment of the densitometer settings until the mean image intensity was centred as nearly as possible at a grey value of 127 on a scale of 0–255.

### 4.3. Image analysis

Micrographs were minimally processed using the ctfit module of EMAN before analysis to correct phase inversion effects [61]. The brightness of entire micrographs was normalized to a common mean value. Data were collected by selecting rectangular regions 8 × 10^−9^ m wide and extending 1.92 × 10^−8^ m above and below the low-density node of the membrane. Linear density traces (figure 3) were calculated by aligning images and averaging the signal from each 8 × 10^−9^ m image row. Small errors in transect centring were corrected by 10 cycles in which density traces were shifted by up to one pixel (4 × 10^−10^ m) to find the alignment with the highest linear correlation to the group average for that round. Electron density peak values were extracted from 2 × 10^−9^ m regions as shown in figure 3.

We examined cryoelectron micrographs of Tacaribe virus to investigate the relationship between protein density and curvature (see the electronic supplementary material, figure S1). Electron density samples were taken from the ‘edge’ with shortest diameter (*d*_min_) where local curvature was lowest, and the ‘tips’ of the particle at the point with the longest diameter (*d*_max_) where local curvature was highest. The virions’ cross sections are approximately elliptical, so these points are an angle *π*/2 apart around the perimeter, i.e. the relevant diameters are perpendicular. Virions were sampled in pairs consisting of one nearly round particle (*d*_max_/*d*_min_ < 1.07) and one elliptical particle (*d*_max_/*d*_min_ > 1.20) of similar size from the same micrograph. Three density samples were taken at each end of *d*_min_, *d*_max_, and at equidistantly spaced points around the perimeter for a total of 24 independent density transects per virion. A total of 7845 data points from 109 particles were used for this analysis (three data points were excluded because measurements were incomplete).

To test the association of viral protein with membrane curvature, a comparison of the (presumed collapsed) relatively flat ‘sides’ of elliptical particles versus their more curved ‘tips’, or the relatively uniform ‘edges’ of almost spherical particles was then undertaken (table 2 and figure 3). To evaluate the role of viral protein in the membrane stiffness, the major and minor axes of each particle, regardless of shape and type, were measured and compared.

### 4.4. Statistical analysis

To evaluate the association of proteins with particle curvature, virions and vesicles were categorized as spherical if the ratio of maximal to minimal axis was less that 1.04, with all other vesicles categorized as elliptical. Measurement points around the circumference of particles were categorized as follows: ‘sides’ of elliptical particles refer to the tangent planes of the minimal axis; ‘tips’ refer to the tangent planes of the maximal axis; ‘edges’ are the equivalent point of relatively uniform circular particles. A mixed effects linear model for the intensity of the signal for each protein (GP, Z and NP) based on position was fitted by restricted likelihood, considering particle and micrograph as nested random effects. The fit in each case was deemed good by inspection of residuals and Q–Q plots. To allow for multiple comparisons, the significance of any difference was evaluated using Tukey's contrast.

To evaluate the magnitude of any potential change in stiffness associated with proteins, the precise shape of vesicles and virions was considered. Because unusually large (or small) virions are significantly less spherical, data here come only from virions (6.0–9.5) × 10^−8^ m in diameter, which covers the majority of particles (51–68% of all present, depending on virus species). Within this range, size is not strongly correlated to shape for PICV (*r* = −0.007), TCRV (*r* = 0.003) or LCMV (*r* = 0.059). By contrast, vesicle shape is unrelated to size over the entire available size range. The deformation ratio *D* (see below) was calculated directly from measurements of *d*_max_ and *d*_min_ using (4.7).

### 4.5. Mechanical membrane model

Motivated by Preston *et al*. [62], the cell membrane is modelled as an area-conserving inextensible thin shell with an intrinsic mean curvature 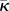, whose resistance to bending is proportional to twice the difference between the actual mean curvature and 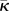. The constant of proportionality is the bending stiffness *B*. We assume that the membrane offers negligible resistance to in-plane shearing, and that the only external forces on it arise from a transmembrane pressure difference *p*.

In the model, budding is driven by changes in 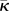 and *B* caused by the presence of an activated protein bound to the inside of the cell membrane. For this initial model, the dynamic interactions with the internal and external cellular fluids are neglected, with the focus on the quasi-steady evolution of the bud as the area *A*_p_ covered by the protein increases.

Outside the budding region, the innate mean curvature 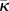 of the membrane is assumed to be a constant 1/*r*_c_ (i.e. in the absence of other forces, the cell would naturally be spherical with radius *r*_c_) and the bending stiffness *B* is assumed to be a constant *B*_0_. In practice, this assumption is only applied to the membrane immediately around the budding region, so the model is equally applicable to deformed and/or non-uniform cells. We assume that the activated protein changes the innate mean curvature to some larger value 1/*ρ* (i.e. the protein-bound membrane would prefer to form a sphere of radius *ρ*) and also alters the bending stiffness to a new value *βB*_0_, where *β* is a constant factor describing the change in stiffness. (We can take *β* = 1 to model no change.)

For the sake of simplicity, it is assumed that the protein forms in an axisymmetric region of area *A*_p_, and that that the bud that forms will also be axisymmetric (i.e. the budding region maintains a circular boundary on the membrane).

To describe the membrane mathematically, we define *s* as the radial arc-length distance along the membrane from the centre of the budding region, and *A*(*s*) as the area of membrane within a distance *s* of the centre. From the assumptions above, we then have that the innate mean curvature is given by4.1
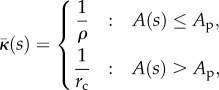
whereas the bending stiffness is given by4.2
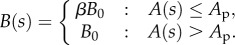
The thin-shell assumption of the model remains valid as long as the radius of curvature of the membrane is large compared with the membrane thickness. Close to pinch-off, the bud develops a very thin and highly curved ‘neck’ region, where this will cease to be true. However, this region is small (both temporally and spatially), and the model does not account for the actual pinching off anyway. We therefore do not consider this to be a significant shortcoming of the model.

The assumption of zero resistance to shear can be justified on two grounds: first that we are considering quasi-static solutions on a slow time-scale over which shear forces can relax. Second, in the model, we use for the membrane mechanics, Preston *et al.* [62] obtain a non-dimensional parameter *C* for the importance of shear relative to bending forces. The equivalent quantity in our notation is 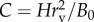. Using the values from table 3, we obtain an approximate range 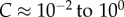. So at least for virions towards the smaller end of the size range in table 3, we also expect transient shear effects to be negligible.

### 4.6. Quantification of cell membrane properties

The estimates we used for the various cell membrane properties are listed in table 3; these were obtained as follows.

The mean radius of mammalian cells *r*_c_ can be measured by direct observation using electron microscopy [63,64], whereas the possible thickness of the cell membrane *δ* is based on a lower estimate of the distance between the inner and outer phosphate groups and an upper bound of the inner and outer edge [65]. The radius of arenavirus virions *r*_v_ has also been measured by electron cryomicroscopy [66].

By contrast, the mechanical properties of cells often need to be derived indirectly. The innate cell membrane bending stiffness *B*_0_ has been estimated experimentally for a number of different bilayers and cell types [67–71], providing a range of values consistent in magnitude. For the membrane shear modulus *H*, we take the range of values given in [70] for red blood cells.

The pressure difference *p* across the cell membrane is dominated by turgor pressure and can therefore vary greatly. In an isotonic state, the osmotic pressure on the cell balances, and the pressure difference is zero by definition. At other times, cells may experience negative pressure in a hypertonic solution (and even undergo pasmosis), or become turgid as a result of a hypotonic solution or cell movement. The latter may result from the formation of a pseudopodium, which can require a significant pressure differential [72] (see also [73]). Although pressure differences of the order of 10 N m^−2^ [73] to 10^2^ N m^−2^ [72] have been recorded, in healthy mammals regulation (by, for example, antidiuretic hormone from the kidneys) ensures that any osmotic imbalance is corrected over a much shorter timescale than that taken for vesicles to form (of the order of minutes). Although it is therefore expected that the pressure differential will be low for much of the time, with cells seeking to be flaccid, this is not assumed to be true in what follows: the only prerequisite on *p* in the model is that it be not too large, in the sense 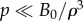, which is satisfied by pressures with magnitudes below *O*(10) N m^−2^ (table 3).

### 4.7. Quantifying the effect of arenavirus proteins

In a process typical of pleomorphic enveloped viruses, arenavirus proteins collect in discrete patches, approximately 200 nm in diameter, at the surface of infected cells [49]. The organization of pre-budding patches closely resembles that of budded virions in cross section, as shown in figure 1. The patches then appear to bend the effectively planar membrane into a virus-sized sphere, which then pinches off from the plasma membrane to form a new virion. Because Z can drive the budding process independently, this suggests that Z has membrane bending and possibly also membrane rigidifying properties.

In our proposed model, it is assumed that membrane curvature is induced by an asymmetric change in the amount of space the GP and Z proteins occupy in the two membrane leaflets. Several cellular [5,10] and viral [12] proteins have been proposed to induce membrane curvature by inserting amphipathic protein domains into one face of the membrane.

The area of the inner membrane initially occupied by a Z-protein is based on the dimensions of the hydrophobic segment embedded in the membrane, derived from the width of the membrane-spanning helix measured from crystal structures [74] and the incremental distance per turn in an α-helix [75].

To estimate the surface density *σ* of Z-proteins, we consider experimental data which suggest that these form groups of two [66] or four [76] proteins, and assume hexagonal packing across the surface of a vesicle. Experimental observations indicate a rhomboidal lattice, which is not inconsistent with this. Using the mean NP spacing distance of 8.5 × 10^−9^ m—considered representative of Z-groups organization [66]—as the side length of a regular hexagon gives a total area of 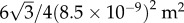. Finally, we note that hexagonal packing implies the area of the hexagon is shared by three protein groups (alternatively, the area each rhombus constructed of two triangles is equivalent to that occupied by a single protein group). So the area occupied by each group is 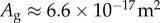. The surface density of Z-proteins (i.e. the number per unit area) is then given by4.3
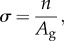
where *n* (equal to two or four) is the number of proteins in a group.

We estimate the value of the protein-induced change in curvature by considering the area of inner cell membrane removed by Z-protein. Consider an initially flat region of cell membrane with thickness *δ* in which both the inner and outer lipid layers have area *A* in the absence of Z-proteins. If each Z-protein removes an area *a* from the inner membrane, then the total area removed is given by *σaA* = *naA*/*A*_g_, assuming all the proteins are triggered. Hence, the relative difference in area between the outer and inner membranes after the Z-protein interactions is given by4.4
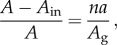
where *A*_in_ is the new area of the inner membrane.

For a membrane with mean curvature *κ* and thickness *δ*, the relative difference in areas between the inner and outer surfaces is given geometrically by 2*κδ* (provided 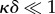, which will be the case here). So, if our membrane adopts its natural mean curvature as induced by the proteins, we will have *κ* = 1/*ρ* and hence4.5
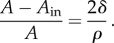


Comparing (4.4) and (4.5), we obtain the estimate4.6
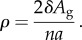


In practice, the existing curvature of the cell means that the inner membrane is already a little smaller than the outer membrane. Thus, (4.6) provides a lower estimate for the innate mean curvature of the bud, and hence upper estimate for *ρ*. But for 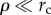 this effect will be small.

Estimates of the likely change in stiffness *β* owing to protein attachment are based on the observed differences in deformation between arenavirus virions and protein-free vesicles released from the same set of cells, i.e. found in the same virus preparations. The latter are made up of cell membrane which appears not to have undergone changes induced by viral protein. We define the relative deformation *D* of a vesicle or virion by4.7
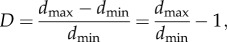
where *d*_max_ and *d*_min_ are the maximum and minimum diameters, respectively.

For vesicles without viral protein the mean value of *D* was 0.070 (based on a total of *n* = 195 vesicles found in the virus preparations). For arenaviruses Pichinde, Tacaribe and LCMV, the mean value of *D* was 0.029, 0.039 and 0.041 (based on *n* = 2810, *n* = 1672 and *n* = 2242 virions), respectively.

For virions and vesicles that exhibit small deformations from a sphere, it is reasonable to suppose that the relative deformation *D* will be inversely proportional to the bending stiffness *B* of the membrane. Assuming that the membranes of the virions have a similar bending stiffness to the cell membrane within the budding area (*B* = *βB*_0_), whereas the vesicles have a similar bending stiffness to the cell membrane outside the budding area (*B* = *B*_0_), we can estimate the stiffness ratio as4.8
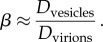


From the data obtained, this provides an estimate of 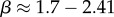.

### 4.8. Model equations

Our equations come from the membrane model derived in [62]. In the interest of brevity, we outline only the key features of the model and the changes we have made here.

**Figure 5.**
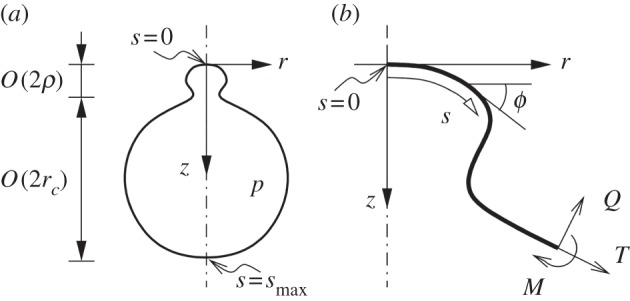
Model coordinate system: (*a*) a sketch of the axisymmetric cell with a virus bud forming near *r* = *z* = 0; (*b*) a close-up of the centre of the budding region, showing the coordinates (*s*,*ϕ*) used describe the membrane in the model. Also shown are the tension *T*, shear force *Q*, moment *M* and boundary conditions.

With the assumed axisymmetric geometry, it is most convenient to describe the membrane shape using an arc-length coordinate *s*, measured from the centre of the bud, and an angle, *ϕ*(*s*), that the membrane surface makes with the horizontal (figure 5).

The internal forces in the membrane are a tension *T*(*s*), a bending moment *M*(*s*) and a shear moment *Q*(*s*). These forces are also depicted in figure 5. The tension is isotropic because of the assumption of zero resistance to shear stress. The bending moment is assumed to be isotropic too, as proposed by Preston *et al*. [62]. Only one component of the shear moment is non-zero, owing to the axisymmetry. The only external force is the transmembrane pressure difference *p*.

Using simple geometry, the radial and vertical distances *r*(*s*) and *z*(*s*) from the centre of the budding region, and also to the area *A*(*s*) enclosed by the circle at *s*, can be related to *s* and *ϕ* by4.9
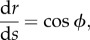
4.10
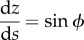
4.11
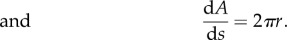
At any given position, the two principal curvatures of the membrane are given by [62]4.12
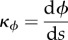
and4.13
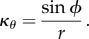
The bending moment *M* generated by the curvature is given by the product of the bending stiffness *B* and the deviation of the total curvature *κ*_*ϕ*_ + *κ*_*θ*_ from the total innate curvature 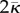:4.14
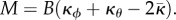


As a result of axisymmetry, the usual six membrane equilibrium equations are reduced to three [62]. We take equations (2.2)–(2.4) of Preston *et al*. [62] and set *N_*ϕ*_* = *N_*θ*_* = *T* (in line with the zero shear resistance assumption), *M_*ϕ*_* = *M_*θ*_* = *M* (in line with the preferred isotropic bending force assumption), and *Q_*ϕ*_* = *Q* (for the sake of simplicity). Then, rearranging (4.14) for *κ*_*ϕ*_ and substituting from (4.13) for *κ*_*θ*_, we obtain4.15
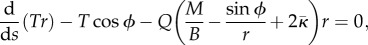
4.16
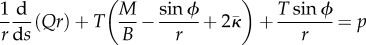
4.17
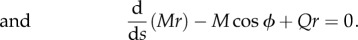
These three equations represent, respectively: the equilibrium balance of forces in the directions of increasing *s*; the equilibrium balance of forces in the normal direction; and the balance of moments acting on a surface element.

Equations (4.9)–(4.11), (4.12) and (4.15)–(4.17) may be recast a set of explicit first-order equations:4.18
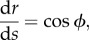
4.19
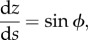
4.20
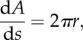
4.21
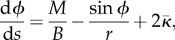
4.22
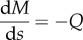
4.23
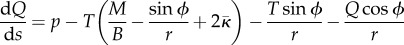
4.24
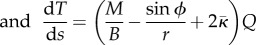
and form a closed seventh-order system for the seven unknowns *r*, *z*, *A*, *ϕ*, *M*, *Q* and *T* as functions of *s*.

### 4.9. Boundary conditions

The equations are to be solved between *s* = 0 at the centre of the bud and *s* = *s*_max_, the as-yet unknown arc-length at the opposite side of the cell (figure 5). (The free parameter *s*_max_ is to be determined as part of the solution.) The boundary conditions at *s* = 0 are4.25
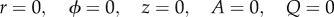
and those at *s* = *s*_max_ are4.26
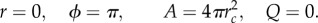
At *s* = 0, we start on the axis (*r* = 0) with the membrane horizontal (*ϕ* = 0). There is no area enclosed between this point and the axis (*A* = 0). By symmetry, we must have *Q* = 0, and we take *z* = 0 to set the origin of the vertical coordinate. At the opposite side of the cell where *s* = *s*_max_, we are again back on the axis (*r* = 0). The membrane is horizontal, but is upside down relative to it orientation at the top (*ϕ* = *π*). The area between this point and *s* = 0 is the full area of the cell membrane, which is fixed and set equal to that of a spherical cell of radius 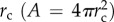. We have *Q* = 0 by symmetry as before, but there is no constraint on *z* at *s* = *s*_max_, because the coordinate origin has already been fixed. (The vertical location of the bottom of the cell is obtained as part of the solution.)

Although there are nine boundary conditions, it is not the case that the seventh-order system is over-determined. The system has a conserved quantity whose value is consistent with both sets of boundary conditions and one free parameter *s*_max_. The conserved quantity arises from a vertical force balance, and is given by4.27
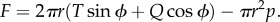


The equations imply d*F*/d*s* = 0, and the boundary conditions at *s* = 0 determine *F* = 0. The conditions at *s* = *s*_max_ are consistent with this, and hence one of them is redundant (although the singularity at *r* = 0 requires the way in which variables approach the limit to be carefully determined). This reduces the effective number of boundary conditions to 8, as required for a seventh-order system with one free parameter.

### 4.10. Non-dimensionalization

Lengths are non-dimensionalized using the natural radius of curvature *ρ* of the budding region, whereas stresses are scaled using *ρ* and the membrane bending stiffness *B*_0_. We define:4.28
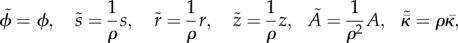
and4.29
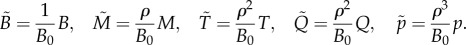
The non-dimensional equations are identical to (4.18)–(4.24), with the addition of tildes and with the innate curvature and bending stiffness now being given by4.30
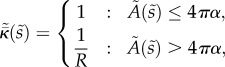
and4.31
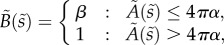
respectively. The dimensionless parameters in the problem of biological relevance are the cell-to-bud size ratio4.32
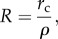
the dimensionless area of the membrane covered by protein4.33
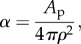
the dimensionless transmembrane pressure difference4.34
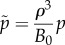
and the stiffness ratio *β*. Finally, 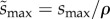 is the dimensionless arc-length at the opposite side of the cell. This is determined as part of the solution, rather than being an input parameter.

Because the budding is driven by a curvature 1/*ρ*, we expect any virions formed to have a radius *r*_v_ of at least *ρ*. Because *r*_v_ is typically much less than the radius *r*_c_ of the cell, this implies 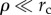, and hence 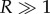. In the absence of any other length scales, we further expect *r_v_* = *O*(*ρ*), and that the area *A*_p_ covered by protein will be roughly 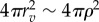. Hence, we expect to need *α* = *O*(1) for bud formation.

### 4.11. Asymptotic solution for small buds (R≫1)

If the bud is small compared with the size of the cell, then away from the budding region, the cell surface is expected to remain spherical to good approximation. We therefore just solve the membrane equations in the neighbourhood of the budding region, and as 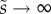 (i.e. as we leave the budding region) the solution must match on to that of a sphere with uniform curvatures4.35
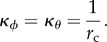
Substituting (4.35) and (4.1) into (4.14) implies that *M* = 0 in the spherical region, from which it follows that *Q* = 0 by (4.22). This implies, by (4.24), that *T* = *T*_0_ is constant. It then follows from (4.23) that the far-field tension *T*_0_ is related to the transmembrane pressure *p* by4.36
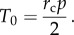
The far-field boundary conditions for the non-dimensional system in the budding region are therefore4.37
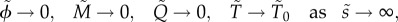
where4.38
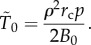
Using (4.34) and (4.38), the non-dimensional transmembrane pressure is then given by4.39
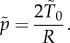
For 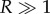, we can neglect the *O*(*R*^−1^) terms in the non-dimensionalized system (4.18)–(4.24). We therefore take4.40
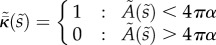
in place of (4.30), and neglect the 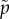 term in (4.23) by virtue of (4.39). (Physically, the latter is equivalent to assuming is that the transmembrane pressure is negligible compared with the large bending forces that arise from the high curvatures in the budding region. This will certainly be the case if 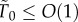.)

With these simplifications, it can be shown that4.41
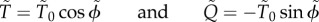
provide exact solutions to equations (4.23) and (4.24) that are consistent with the boundary conditions (4.25) at 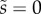 and (4.37) as 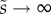. The system of interest then reduces to4.42
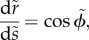
4.43
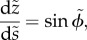
4.44
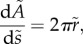
4.45
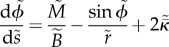
4.46
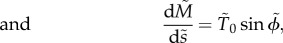
with 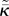 and given by (4.40) and (4.31), respectively. The boundary conditions at infinity reduce to 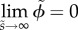, but numerically the boundary conditions must be applied at a large but finite value of 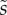. Hence, we impose4.47
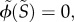
where 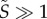 is a constant.

Because the equations are singular at 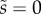, the boundary conditions (4.25) at the origin cannot be imposed directly in a numerical scheme. A series solution of (4.42)–(4.46) about 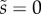 is required. For 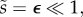 we find that (4.25) implies4.48
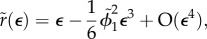
4.49
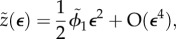
4.50
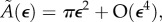
4.51
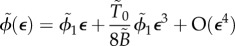
4.52
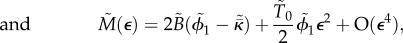
where 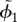 is an unknown constant.

Equations (4.42)–(4.46) constitute a fifth-order system with one unknown parameter 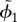, subject to six boundary conditions (4.47)–(4.52). Here, 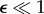 and 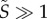 are artificial numerical parameters, whose exact values should not affect the solution. The biological parameters *β* and 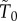 are set by the cell and virus properties, whereas *α* is the non-dimensional area of the cell membrane covered by protein.

The energy stored in the membrane owing to the deviation from its natural curvature is given by4.53
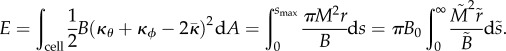
The second equality comes from using (4.14) to eliminate the curvatures in favour of *M*, and using (4.20) to perform a change of variables from *A* to *s*. The final equality comes from using the non-dimensionalization (4.28) and (4.29).

### 4.12. Numerical methods

The Matlab routine bvp4c (implementing the three-stage Lobatto IIIa formula) was used to solve the two-point boundary-value problem (4.42)–(4.46) for 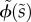 and 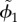, subject to (4.48)–(4.52) at 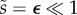 and (4.47) at 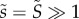. Numerical results were verified by comparison with a C++ shooting program that implements Runge–Kutta integration and Newton's method (based on algorithms from *Numerical recipes* [77]) and integration with ode45 in Matlab.

Condition (4.47) is applied at a large value 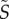 of 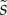: in practice, 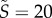 proved more than sufficient for far-field behaviour to become clear. Near the origin, 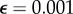 proved sufficiently small for accurate results. The solution of the system is not unique, although we ever found only one physically appropriate solution for any set of parameters 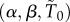. (Other solutions all resulted in self-intersecting membrane curves, and so had to be rejected.) The appropriate solution is tracked through 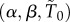 parameter space by changing the relevant parameters incrementally and applying previous solution for 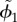 as the initial condition for subsequent numerical estimates.

The relative length scales of the problem, and model dynamics, are determined by the parameters of the system. These are taken from the literature—see §§4.6 and 4.7 for full details; the estimated (range of) values for each parameter are given in table 3.
